# Head-to-head comparison of [^18^F]-mFBG versus [^123^I]-MIBG in neuroblastoma: a systematic review and meta-analysis

**DOI:** 10.1007/s00247-026-06713-z

**Published:** 2026-07-08

**Authors:** Domenico Albano, Francesco Bertagna, Arnoldo Piccardo, Francesco Fiz, Giorgio Treglia

**Affiliations:** 1 University of Brescia, Brescia, 25121 Italy; 2 Spedali Civili di Brescia, Brescia, Italy; 3 Ente Ospedaliero Ospedali Galliera, Genoa, Italy; 4 Ospedale Regionale di Bellinzona e Valli, Bellinzona, Switzerland

**Keywords:** PET/CT, Neuroblastoma, MIBG, MFBG, Nuclear medicine, SPECT/CT

## Abstract

Hybrid imaging is fundamental in the staging and response assessment of patients affected by neuroblastoma (NB). [^123^I]-metaiodobenzylguanidine (MIBG) scintigraphy±single-photon emission computed tomography/computed tomography (SPECT/CT) is considered the gold standard for the evaluation of NB, but it presents some limitations, such as the time-consuming procedure (2 days), the low spatial resolution, and challenging interpretation. Alternative positron emission tomography/computed tomography (PET/CT) catecholaminergic radiopharmaceuticals have been investigated to overtake the issues of [^123^I]-MIBG. Among them, one of the most promising seems to be meta-[^18^F]fluorobenzylguanidine ([^18^F]-mFBG). Thus, we planned a head-to-head systematic review and meta-analysis to compare [^18^F]-mFBG PET/CT and [^123^I]-MIBG SPECT/CT in NB. We executed a literature search to identify all articles performing a head-to-head comparison between these two methods, excluding review articles, preclinical studies, case reports, and small case series (less than 5 patients). Finally, five studies were collected for a total of 115 patients and 1,644 lesions. A patients-based analysis showed that [^18^F]-mFBG PET/CT had a higher pooled sensitivity than [^123^I]-MIBG SPECT/CT (97% vs 82%, respectively) and identical pooled specificity (82%). Also at a lesion-based analysis, [^18^F]-mFBG PET/CT demonstrated better sensitivity (99% vs 51.5%). In conclusion, preliminary evidence-based data demonstrate that [^18^F]-mFBG PET/CT showed superior diagnostic performance than [^123^I]-MIBG SPECT/CT. However, it is still unclear the real added value of [^18^F]-mFBG PET/CT compared to [^123^I]-MIBG SPECT/CT in the change of clinical management. These findings suggest [^18^F]-mFBG PET/CT may become a superior, more efficient alternative to [^123^I]-MIBG for staging and response assessment in neuroblastoma. Future studies should focus on its impact on clinical management and patient outcomes.

**Graphical Abstract:**

**Figure:**
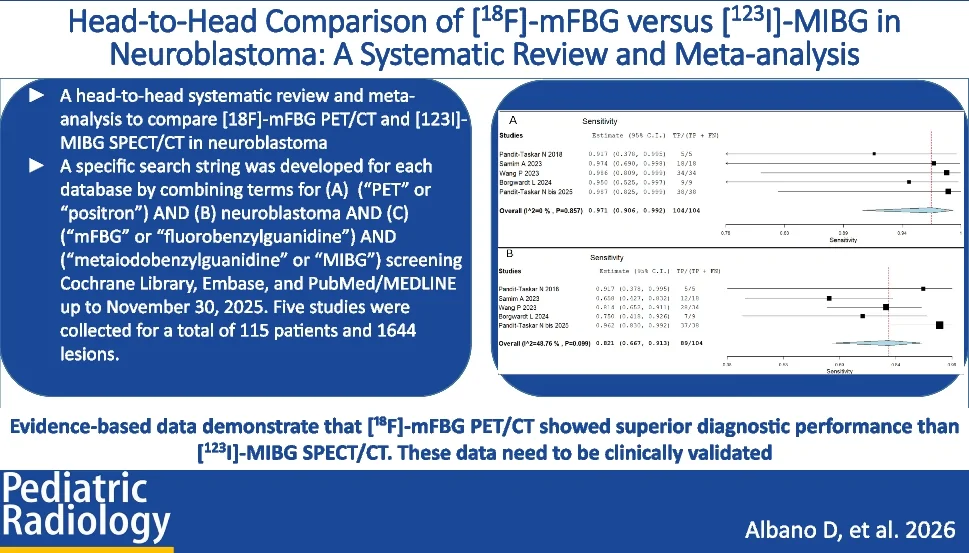

**Supplementary Information:**

The online version contains supplementary material available at https://doi.org/10.1007/s00247-026-06713-z.

## Introduction

Neuroblastoma (NB) is a rare disease and the most common extracranial pediatric solid tumor that originates from the sympathetic nervous system and often results in an overproduction of catecholamines. For patients with high-risk disease, the presence of metastases at diagnosis leads to a poor prognosis, with long-term survival rates hovering around 40–50% [1, 2].

Therapy of NB requires intensive, multi-step regimens including chemotherapy, surgery, high-dose chemotherapy with stem cell support, and various forms of radiation and targeted therapies, like external beam radiotherapy and anti-GD2-directed immunotherapy [3]. An accurate diagnostic assessment before treatment seems to be crucial.

Hybrid imaging is paramount for staging and evaluating treatment response in NB. The current gold standard molecular imaging method is considered [^123^I]-MIBG whole-body scintigraphy, often combined with tomographic acquisitions (SPECT/CT) [4]. [^123^I]-MIBG is a radiolabeled aralkylguanidine analog of the adrenergic neurotransmitter norepinephrine; it localizes to adrenergic tissue and thus can be used to identify neural crest tumors such as NB. This tool is highly effective for detecting NB lesions including distant metastases and offers significant predictive and prognostic value through a validated scoring system [5, 6]. However, [^123^I]-MIBG imaging has several limitations. First, it can provide false negative results in 10% of patients [7, 8]; second, this examination has suboptimal spatial resolution compared to cross-sectional imaging; third, it is a time-consuming imaging consisting of multi-day protocol and requiring careful thyroid blockade. Thus, alternative nuclear medicine methods including the use of novel positron emission tomography (PET) tracers have been investigated and tested to overcome the limitations of [^123^I]-MIBG imaging. These catecholaminergic tracers, such as meta-hydroxyephedrine ([^11^C]C-HED), ^18^F-18F-3,4-dihydroxyphenylalanine ([^18^F]-DOPA) [^124^I]mIBG and meta-[^18^F]fluorobenzylguanidine ([^18^F]-mFBG), offer the potential to assess the catecholaminergic pathway, and showed good diagnostic performances and a positive impact in the management of NB patients [9, 10].

Even if some original studies have provided high detectability of [^18^F]-mFBG PET/CT compared to [^123^I]mIBG results, a pooled analysis of available literature data through a systematic review and a head-to-head comparison, increasing the statistical power, could provide more solid data than original studies about the comparison among these imaging methods [11].

Therefore, we performed a systematic review and meta-analysis with the primary aim of comparing the diagnostic performance (sensitivity and specificity) of [^18^F]-mFBG PET/CT and [^123^I]-MIBG SPECT/CT in neuroblastoma patients, both on a per-patient and per-lesion basis. A secondary aim was to summarize the technical aspects and study characteristics of the available literature.

## Methods

### Protocol

The systematic review and meta-analysis was conducted and reported following the Preferred Reporting Items for Systematic Reviews and Meta-Analyses (PRISMA) guidelines [12]. The full PRISMA checklist is available in the Supplemental Fig. 1.

**Fig. 1:**
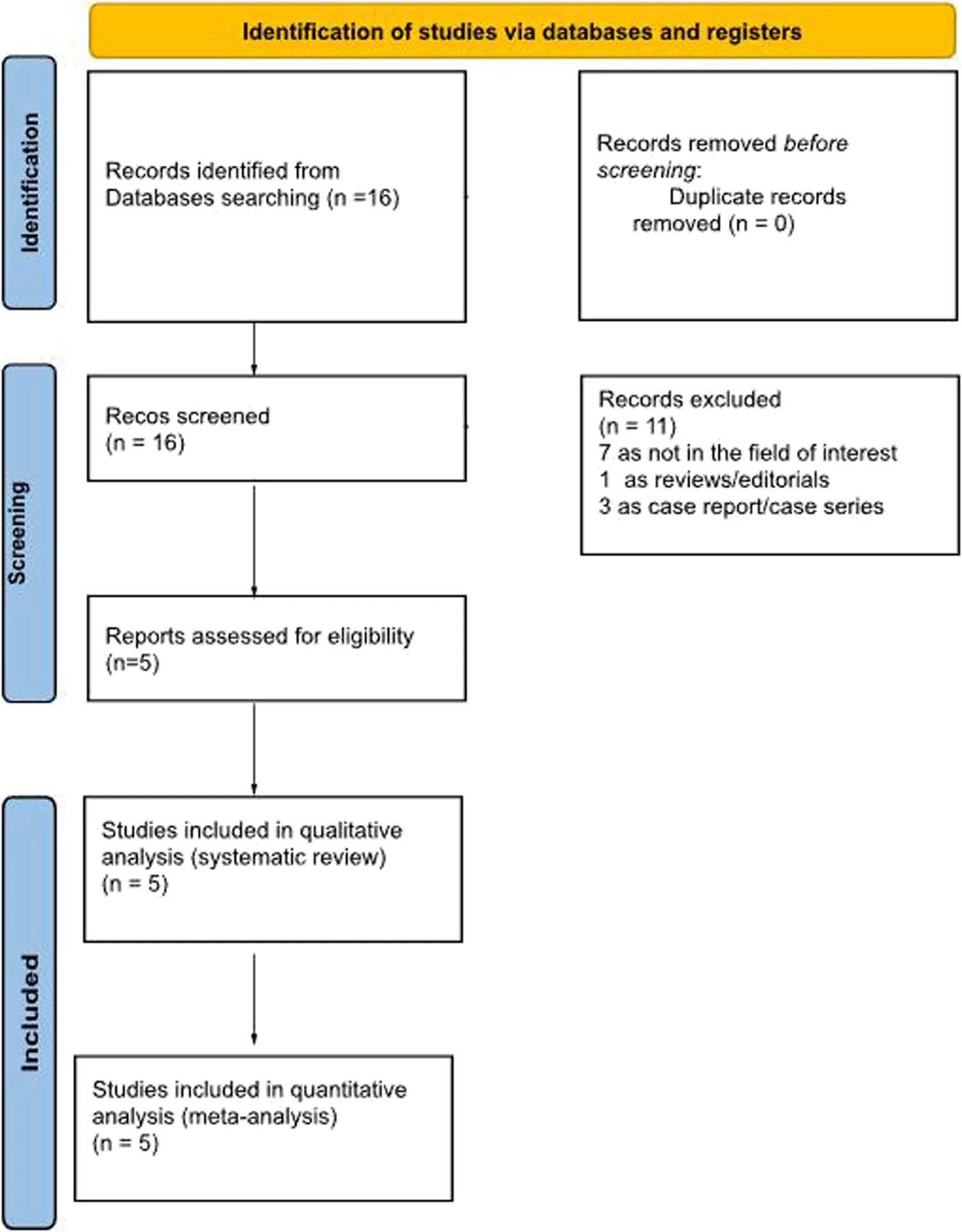
Preferred reporting items for systematic reviews and meta-analyses flowchart [12]

### Search strategy

The search strategy involved two nuclear medicine physicians (D.A. with more than 10 years of experience and G.T. with more than 20 years) independently screening Cochrane Library, Embase, and PubMed/MEDLINE up to November 30, 2025. A specific search string was developed for each database by combining terms for (A) (“PET” or “positron”) AND (B) neuroblastoma AND (C) (“mFBG” or “fluorobenzylguanidine”) AND (“metaiodobenzylguanidine” or “MIBG”). To further enhance sensitivity, reference lists of potentially relevant studies were also manually examined. No language or publication date restrictions were applied. All initially eligible articles were screened, and only those reporting a head-to-head comparison of [^18^F]-mFBG PET/CT and [^123^I]-MIBG in at least 5 NB patients were included. Review articles, studies based on preclinical data, phantom studies, case reports, small case series, and studies with overlapping data were excluded.

### Data extraction and collection

Data extraction was performed independently by two authors (D.A and G.T.) from the full-text articles and their supplementary materials. The following details were collected:

- Studies overview: publication details (authors, year, country), study design, funding sources, population features (age, gender);
- Imaging parameters: kind of device, mean injected activity, uptake time, the interval between the two imaging procedures, image analysis methods;
- Diagnostic performances expressed as sensitivity and specificity of both imaging procedures, calculated using a patient-based analysis (PBA) and a lesion-based analysis (LBA) for each study, if possible;
- Validation standard: the standard of reference utilized.

If some required data were absent, the corresponding authors were contacted by email. A thorough cross-checking of the extracted data was performed, and any resulting disagreements were reconciled through a joint consensus meeting.

### Quality assessment (risk of bias assessment)

The risk of bias and applicability concerns for all included studies were assessed by one author (G.T.) using the Quality Assessment of Diagnostic Accuracy Studies-2 (QUADAS-2) tool [13]. This tool comprises four domains: Patient Selection, Index Test, Reference Standard, and Flow & Timing. Each domain was scored as having a “high,” “low,” or “unclear” risk of bias. For the purpose of a quantitative summary score, “high” risk was assigned 1 point and “unclear” risk 0.5 points. A study with a total score ≥4 across all domains was considered to have an unacceptable risk of bias, but none of the included studies met this exclusion threshold.

To minimize the risk of circular validation, the reference standard was defined as a composite of all available data, including cross-sectional imaging (CT or MRI), laboratory findings and clinical/radiological follow-up. While [^123^I]-MIBG was part of the initial workup, the “truth” was confirmed by the consensus of these multiple independent parameters.

### Statistical analysis

Diagnostic accuracy metrics were derived from each study by performing a PBA and LBA that determined the number of True Positive (TP), False Positive (FP), True Negative (TN), and False Negative (FN) findings. The primary outcome measures for the quantitative synthesis were pooled sensitivity and specificity. These were calculated using a bivariate random-effects model, a statistical approach specifically chosen because it accounts for the potential correlation between sensitivity and specificity. All pooled outcomes are reported with their respective 95% confidence interval (95% CI) values. A summary receiver operating characteristic (SROC) curve correlating sensitivity to specificity was also provided to summarize the diagnostic performance of the index test. The presence and extent of heterogeneity were assessed using the *I*-squared (*I*^2^) index, with values over 50% indicating substantial heterogeneity. Statistical analysis was conducted using the open-source software OpenMeta Analyst® (Brown University, Providence, RI, version 10.12).

## Results

### Literature search

Our initial literature search across the three selected databases yielded 16 records (Fig. 1). We then screened these articles, starting with the review of titles and abstracts. This initial pass led to the exclusion of 11 articles for several reasons: 7 were outside our specific field of interest, 3 were either small case series or individual case reports, 1 was identified as review. Finally, 5 studies [14–18] were deemed relevant enough for a thorough full-text review and were subsequently included in this systematic review. A final check of the references within these selected articles did not reveal any new records for inclusion.

### Quality assessment

**Fig. 2:**
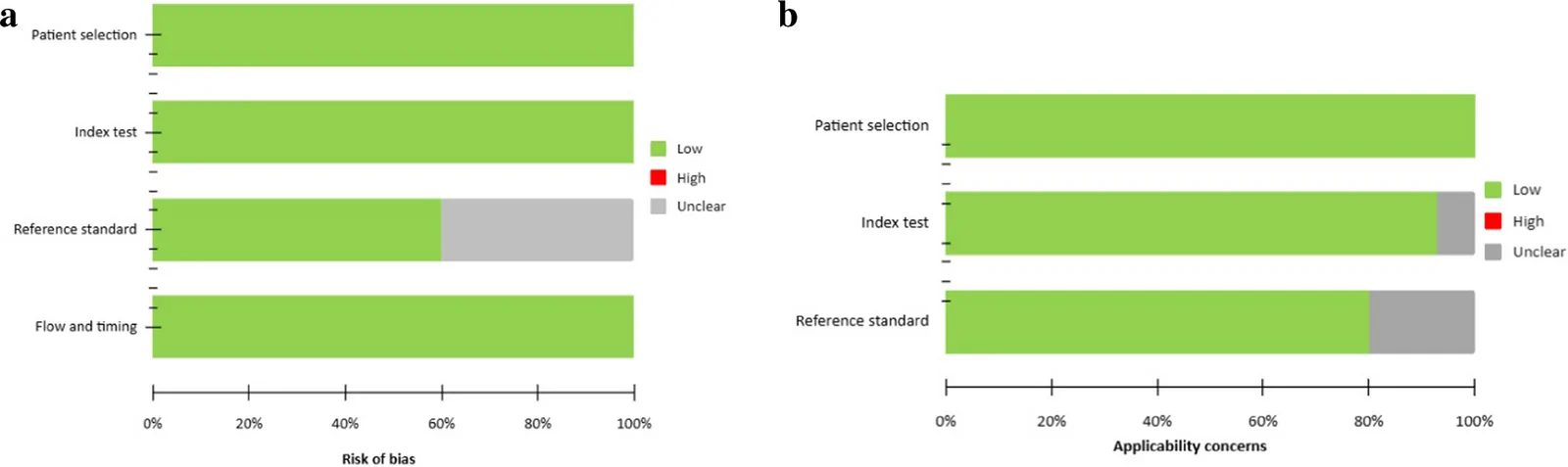
Summary of the quality assessment of the included studies using the QUADAS-2 tool divided for the risk of bias (**a**) and applicability concerns (**b**)

The risk of bias was assessed according to seven items and represented in Fig. 2. No studies were excluded because of high bias risk. The most frequent sources of possible bias were the “study test” and “reference standard categories” since, in some studies, it was unclear whether a blinded evaluation of the two methods had been performed and which gold standard was considered for the final diagnosis.

### Studies and patients characteristics

Table 1 details the key characteristics of the articles included in our qualitative analysis. All the studies were of prospective nature and were published after 2018 in the USA (*n*=2), the Netherlands (*n*=1), Denmark (*n*=1), and China (*n*=1). Funding sources were reported in all studies.

**Table 1:** Main features of the studies selected

First author	Year	Country	Funding source	Study design	No. pts (no. scans)	M:F	Average age (range)	Interval between [^123^I]-MIBG and [^18^F]-FDG, days	Reference standard
Pandit-Taskar N [14]	2018	USA	Yes	P	5*	3:2	5–23	na	Standard imaging consensus (CT/MRI/MIBG)
Samim A [15]	2023	Netherlands	Yes	P	14 (20)	6:8	4.9 (0.2–16)	3.5 (1–9)	Standard imaging consensus (CT/MRI/MIBG)
Wang P [16]	2023	China	Yes	P	40 (40)	nr	6 (1.5–17)	Within 1 week	Standard imaging consensus (CT/MRI/MIBG)
Borgwardt L [17]	2024	Denmark	Yes	P	10 (10)	nr	1.6 (0.1–7.9)	Within 1 week	Standard imaging consensus (CT/MRI/MIBG)
Pandit-Taskar N [18]	2025	USA	Yes	P	37 (40)	20:17	4–45	4.65 (0–28)	Standard imaging consensus (CT/MRI/MIBG)

*M* male, *F* female, *R* retrospective, *P* prospective*, na* not available, *CT* computed tomography, *MRI* magnetic resonance imaging

^*^5 other patients with paraganglioma or pheochromocytoma

There was a balance concerning gender ratio; age ranged from 0.1 months to 45 years.

In all studies the standard imaging of the final diagnosis was not histological diagnosis but imaging findings, derived by a combination of CT, MRI, and scintigraphy reports.

A critical point of methodological heterogeneity across all studies is the reference standard. None used histopathological confirmation for all lesions, which represents an intrinsic limitation. Instead, the final diagnosis was based on a consensus of standard imaging (CT, MRI, and [^123^I]-MIBG itself), potentially introducing incorporation bias, as the index test ([^18^F]-mFBG) was compared against a standard that included the comparator ([^123^I]-MIBG). This may overestimate the specificity of both tests and limits the strength of conclusions regarding true diagnostic accuracy.

### Technical aspects

The imaging modality consisted of hybrid PET/CT (for [^18^F]-mFBG) and planar imaging plus SPECT/CT (for [^123^I]-MIBG) with low-dose computed tomography in all cases, except one with mixed PET/CT and PET/MRI [18].

The injected radiotracer activity ranged from 148 MBq to 444 MBq as absolute value or 1.5–3 MBq/kg for [^18^F]-mFBG PET/CT and from 80 MBq to 436 MBq as absolute value or 4–5.18 MBq/kg for [^123^I]-MIBG. The time interval between [^18^F]-mFBG injection and PET/CT image acquisition was similar across the studies, being 60 min in the majority of cases but one [14] in which PET/CT acquisition was performed with dual-time protocol (60–120 min and 180–240 min after the radiotracer injection). Concerning [^123^I]-MIBG, only one acquisition at 24 h was present. Only in one study, a 2-day protocol was performed [14].

In all PET studies, images were analyzed by a combination of visual (qualitative) and semiquantitative analyses, the latter using the maximum standardized uptake values (SUVmax) as the most frequent variable measured. Concerning SPECT/CT, only visual analysis was performed in all cases (Table 2).

**Table 2:** Technical characteristics of studies included

First author	[^18^F]mFBG				[^123^I]-MIBG			
	Device	Mean injected dose, MBq	Mean uptake time (min)	Image analysis	Device	Mean injected dose, MBq	Mean uptake time (h)	Image analysis
Pandit-Taskar N [14]	PET/CT Discovery 710 (GE)	148–444	60–120, and 180–240	Visual and semiquantitative analysis (SUVmax)	SPECT/CT model not reported	162–436	6 and 24	Visual
Samim A [15]	PET/CT Biograph vision 600 (Siemens)	2/kg	60	Visual (SIOPEN score) and semiquantitative analysis (SUVmax, SUVmean)	Symbia Intevo 16 Bold SPECT/CT (GE)	4/kg	24	Visual (SIOPEN score)
Wang P [16]	PET/CT Polestar m660 (SinoUnion)	2–3/kg	90	Visual (Curie score) and semiquantitative analysis (SUVmax, TBR)	64-slice Philips Precedence device SPECT/CT (Philips)	5.18/kg	20–24	Visual (Curie and SIOPEN score)
Borgwardt L [17]	PET/CT Biograph Vision Quadra (Siemens)	1.5–3/kg	60	Visual (Curie and SIOPEN score) and semiquantitative analysis (SUVmax, SUV ratio liver)	Symbia Intevo 16 Bold SPECT/CT (Siemens)	80–200	24	Visual (Curie and SIOPEN score)
Pandit-Taskar N [18]	PET/CT Discovery 710 (GE) or PET/MR Signa (GE)	444	60	Visual (Curie score) and semiquantitative analysis (SUVmax)	SPECT/CT model not reported	5.18/kg	24	Visual (Curie score)

*na* not available, *SUV* standardized uptake value, *TBR* tumor-to-background ratios

### Diagnostic performances

Finally, five studies were collected for a total of 115 patients and 1,644 lesions. The five articles selected included populations consisting of 5 to 40 patients affected by NB.

**Fig. 3:**
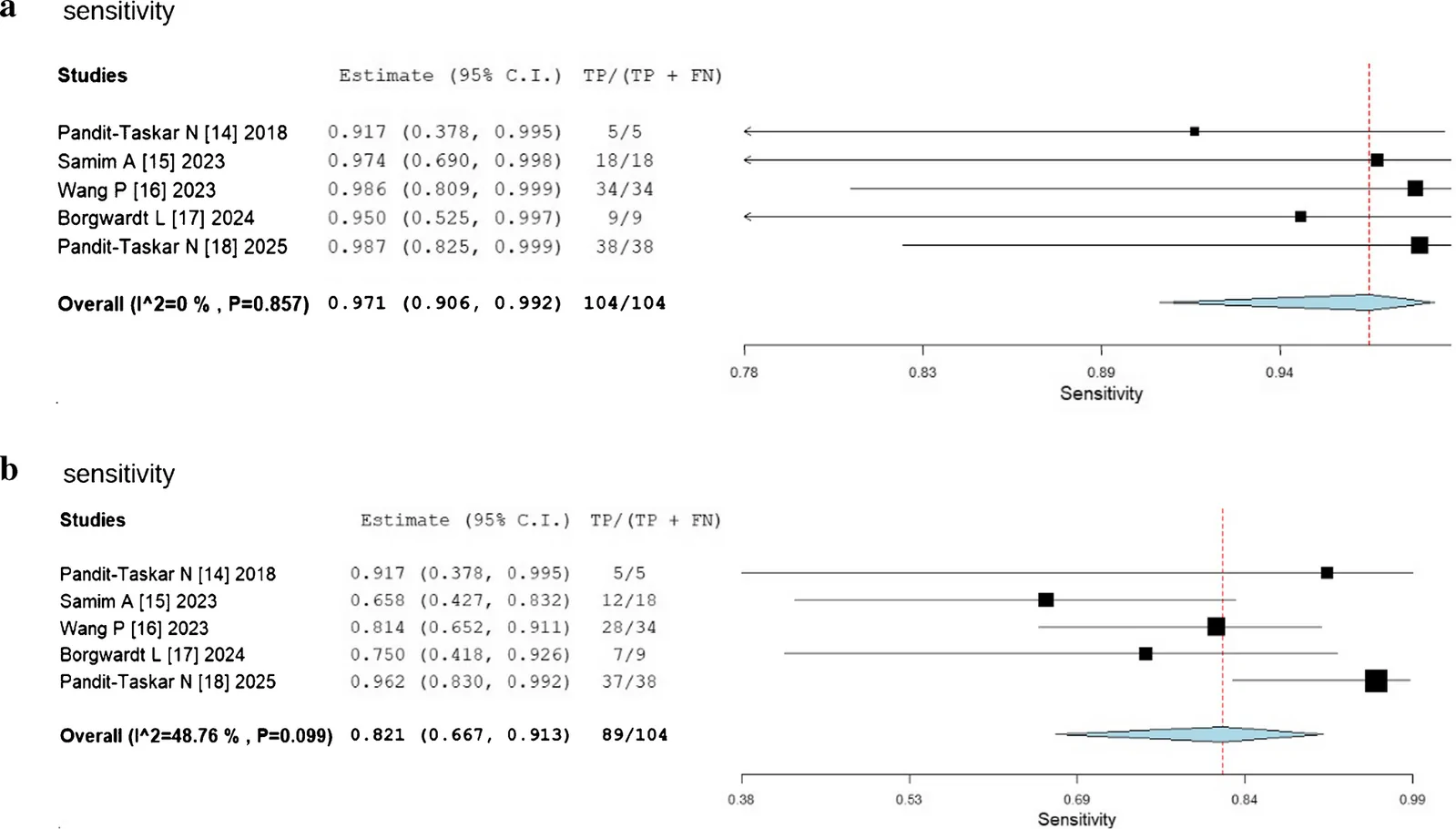
Forest plots of individual studies and sensitivity of [^18^F]-mFBG PET/CT (**a**) and [^123^I]-MIBG SPECT/CT (**b**) in neuroblastoma in a PBA. The size of the *squares* shows the weight of every article. The *horizontal lines* represent the 95% CI values for every research, whereas the *horizontal diameter* of the rhombus represents the 95% CI value for the pooled sensitivity

A PBA demonstrated that [^18^F]-mFBG PET/CT had better diagnostic performances than [^123^I]-MIBG SPECT/CT in four articles [15–18] and equal in the remaining [14] (Table 3). The pooled sensitivity of [^18^F]-mFBG PET/CT was 97% (95% CI 91–99%) compared to 82% (95% CI 67–91%) for [^123^I]-MIBG SPECT/CT (Fig. 3).

**Table 3:** Diagnostic performance of ^[18]^F-mFBG and [^123^I]-MIBG in a per-patient analysis

First author	Year	[^18^F]mFBG				[^123^I]-MIBG			
		TP	FP	FN	TN	TP	FP	FN	TN
Pandit-Taskar N [14]	2018	5	0	0	0	5	0	0	0
Samim A [15]	2023	18	0	0	2	12	0	6	2
Wang P [16]	2023	34	0	0	6	28	0	6	6
Borgwardt L [17]	2024	9	0	0	1	7	0	2	1
Pandit-Taskar N [18]	2025	38	0	0	2	37	0	1	2
Global		104	0	0	11	89	0	15	11

*TP* true positive, *TN* true negative, *FP* false positive, *FN* false negative, *na* not available

No statistically significant heterogeneity among the studies was found for both tracers (*I*^2^<50%).

**Fig. 4:**
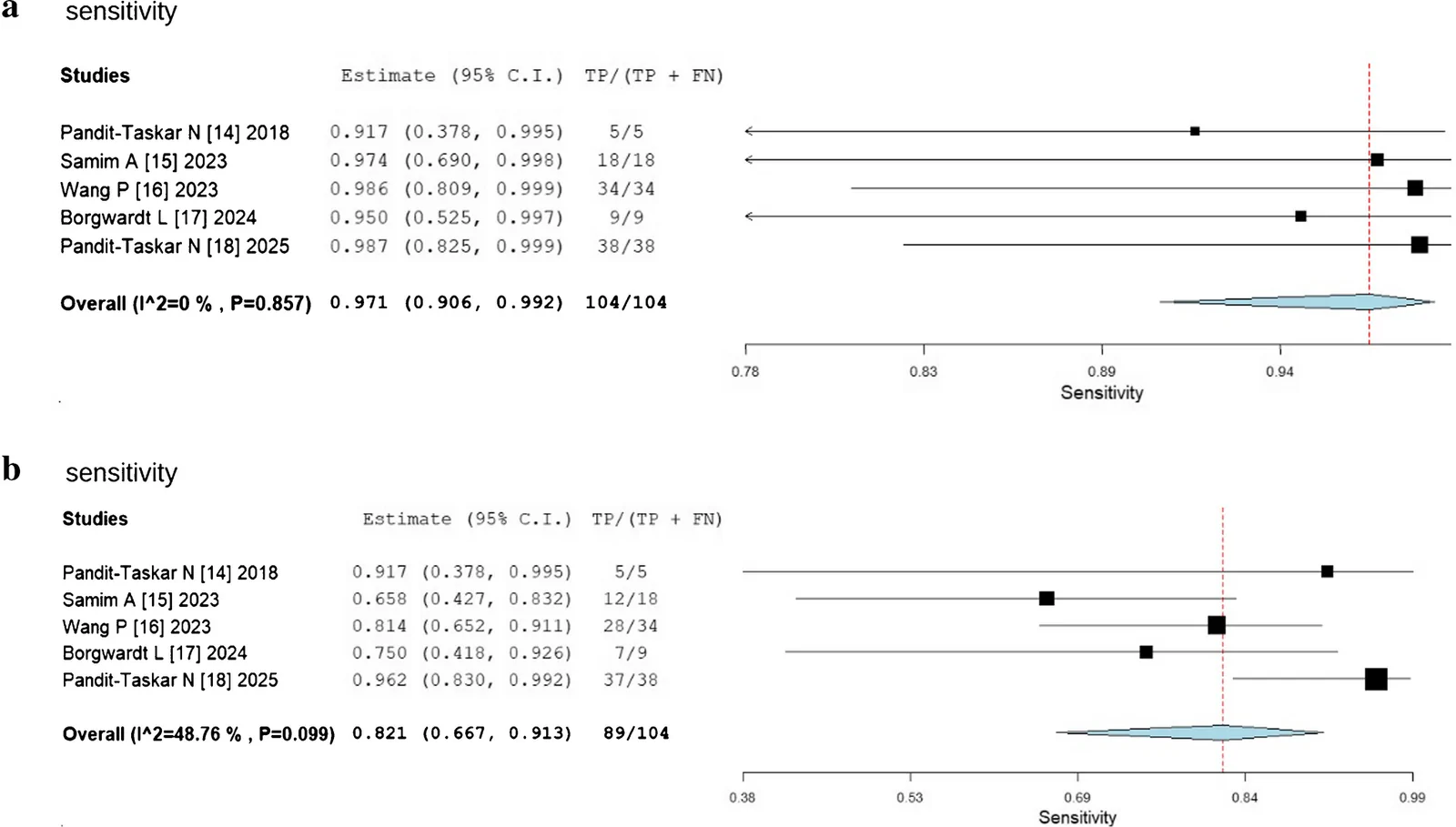
Forest plots of individual studies and specificity of [^18^F]-mFBG PET/CT (**a**) and [^123^I]-MIBG SPECT/CT (**b**) in neuroblastoma in a PBA. The size of the *squares *shows the weight of every article. The *horizontal lines* represent the 95% CI values for every research, whereas the *horizontal diameter* of the rhombus represents the 95% CI value for the pooled specificity

Concerning PBA specificity, the performances of the two methods were the same: 82% (95% CI 53–95%) (Fig. 4).

**Fig. 5:**
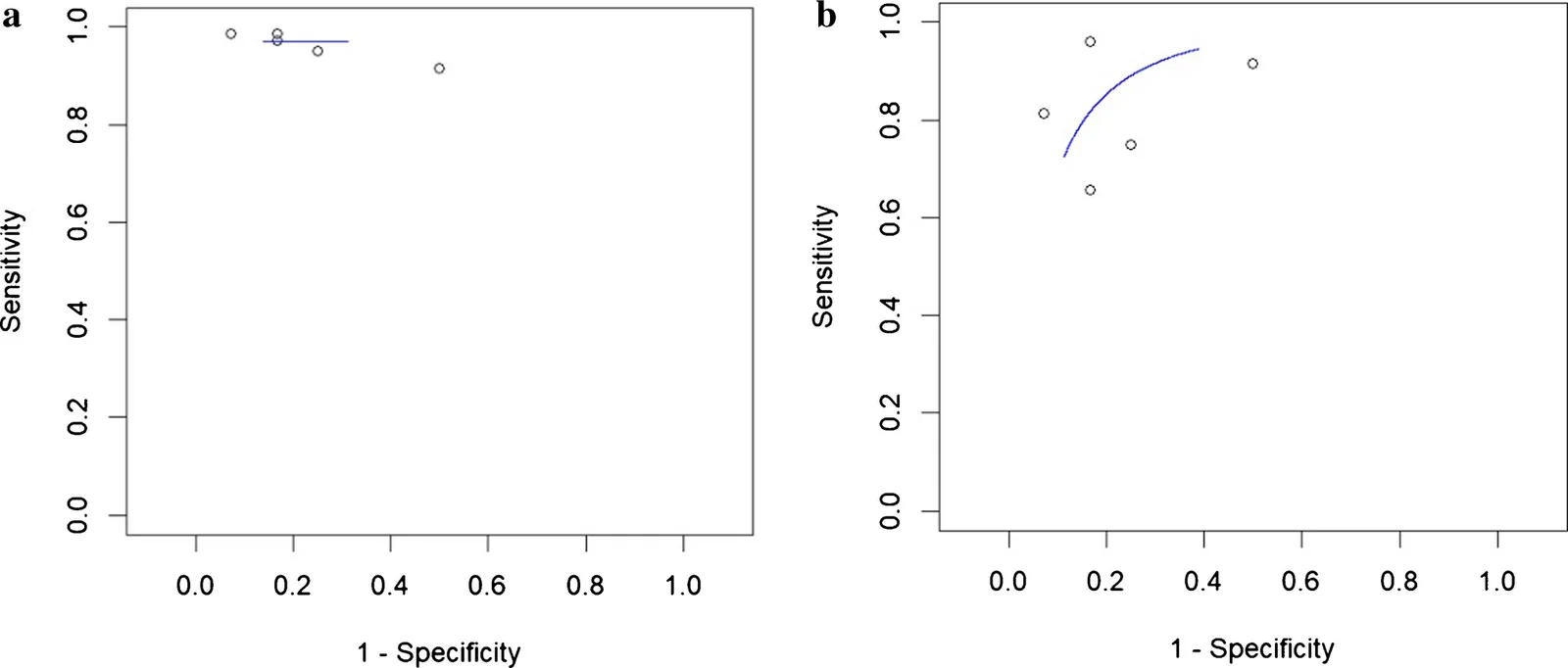
Summary receiver operating characteristics curve on the diagnostic performance of [^18^F]-mFBG PET/CT (**a**) and [^123^I]-MIBG SPECT/CT (**b**) in neuroblastoma on patient based analysis

SROC curves are represented in Fig. 5 and demonstrated an optimal diagnostic performance of [^18^F]-mFBG PET/CT and a good diagnostic performance of [^123^I]-MIBG SPECT/CT.

**Fig. 6:**
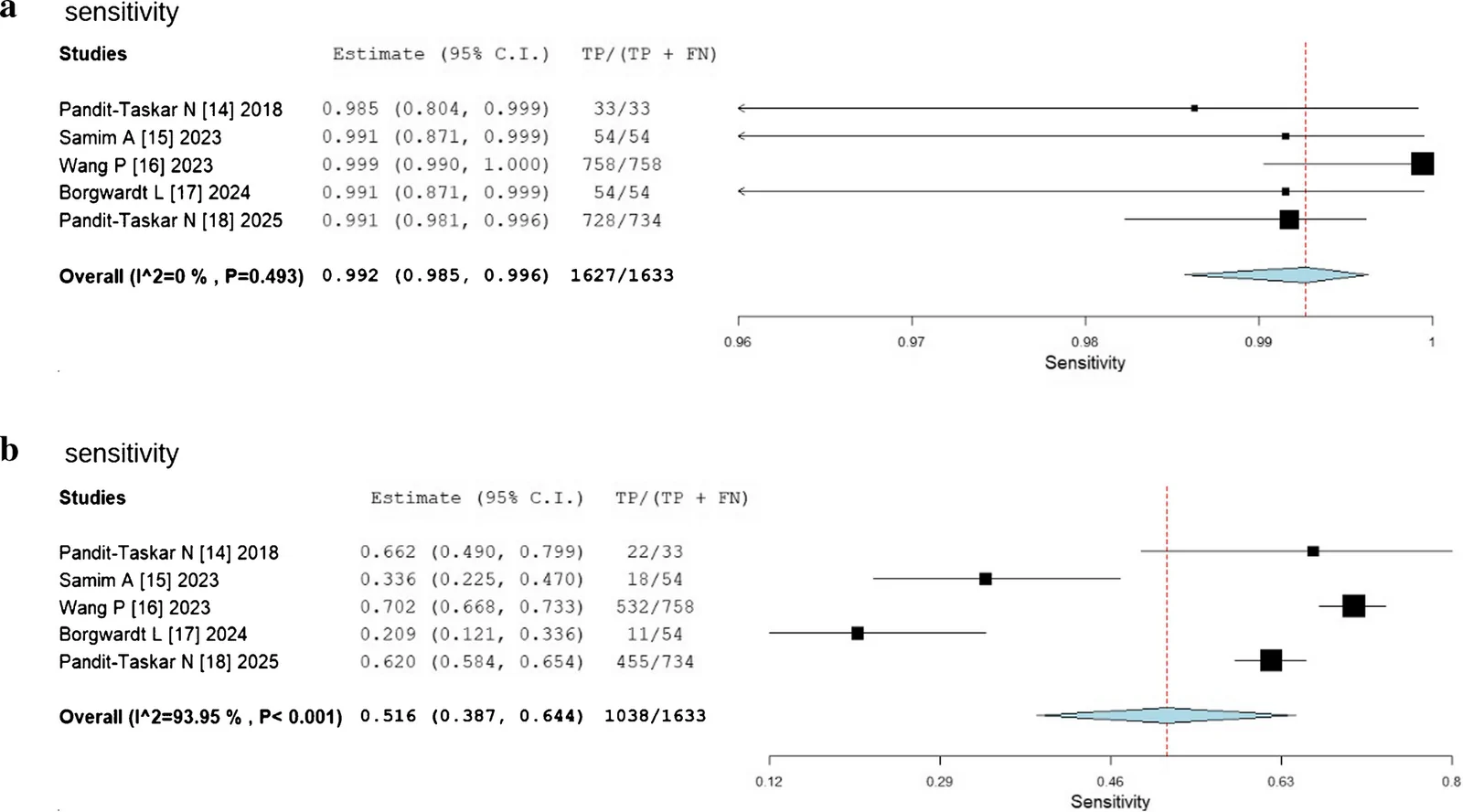
Forest plots of individual studies and sensitivity of [^18^F]-mFBG PET/CT (**a**) and [^123^I]-MIBG SPECT/CT (**b**) in neuroblastoma in a lesion based analysis. The size of the squares shows the weight of every article. The *horizontal lines* represent the 95% CI values for every research, whereas the *horizontal diameter* of the rhombus represents the 95% CI value for the pooled sensitivity

**Fig. 7:**
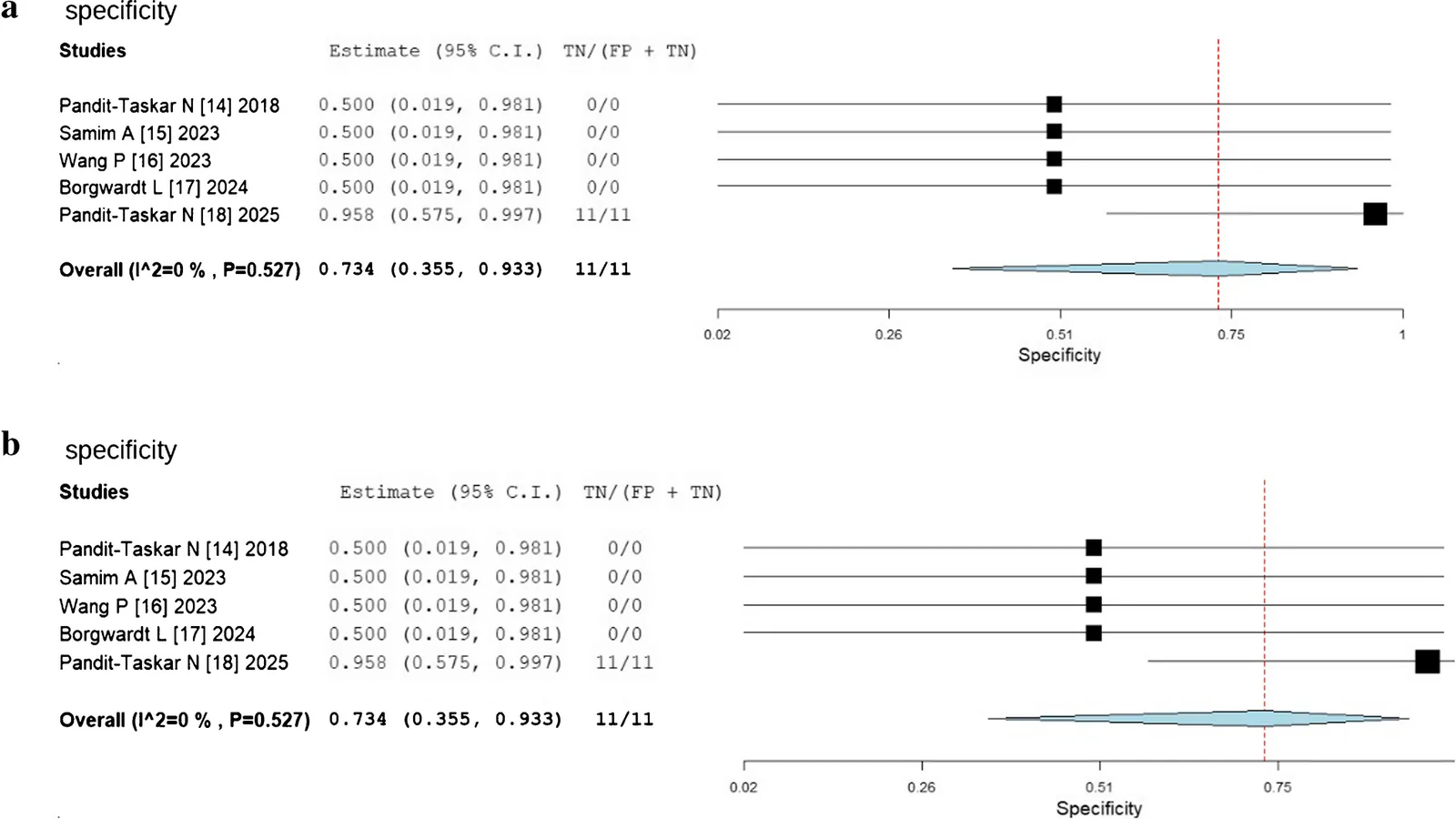
Forest plots of individual studies and specificity of [^18^F]-mFBG PET/CT (**a**) and [^123^I]-MIBG SPECT/CT (**b**) in neuroblastoma in a lesion based analysis. The size of the squares shows the weight of every article. The *horizontal lines* represent the 95% CI values for every research, whereas the *horizontal diameter* of the rhombus represents the 95% CI value for the pooled specificity

About LBA, considering a total of 1,644 lesions, the difference between [^18^F]-mFBG PET/CT and [^123^I]-MIBG SPECT/CT was more significant (Table 4). The pooled sensitivity and specificity of [^18^F]-mFBG PET/CT were 99.2% (95% CI 98.5–99.6%) and 73% (95% CI 36–93%), respectively, without significant heterogeneity (*I*^2^=0%), while for [^123^I]-MIBG SPECT/CT, the same values were 51.5% (95% CI 39–64%) and 73% (95% CI 36–93%), respectively (Figs. 6 and 7). Heterogeneity for sensitivity of [^123^I]-MIBG SPECT/CT was very high (*I*^2^=94%), while not significant for specificity (*I*^2^=0).

**Table 4:** Diagnostic performance of ^[18]^F-mFBG and [^123^I]-MIBG in a per-lesion analysis

First author	Year	[^18^F]mFBG				[^123^I]-MIBG			
		TP	FP	FN	TN	TP	FP	FN	TN
Pandit-Taskar N [14]	2018	33	0	0	0	22	0	11	0
Samim A [15]	2023	54	0	0	0	18	0	36	0
Wang P [16]	2023	758	0	0	0	532	0	226	0
Borgwardt L [17]	2024	54	0	0	0	11	0	43	0
Pandit-Taskar N [18]	2025	728	0	6	11	455	0	279	11
Global		1,627	0	6	11	1,038	0	595	11

*TP* true positive, *TN* true negative, *FP* false positive, *FN* false negative, *na* not available

**Fig. 8:**
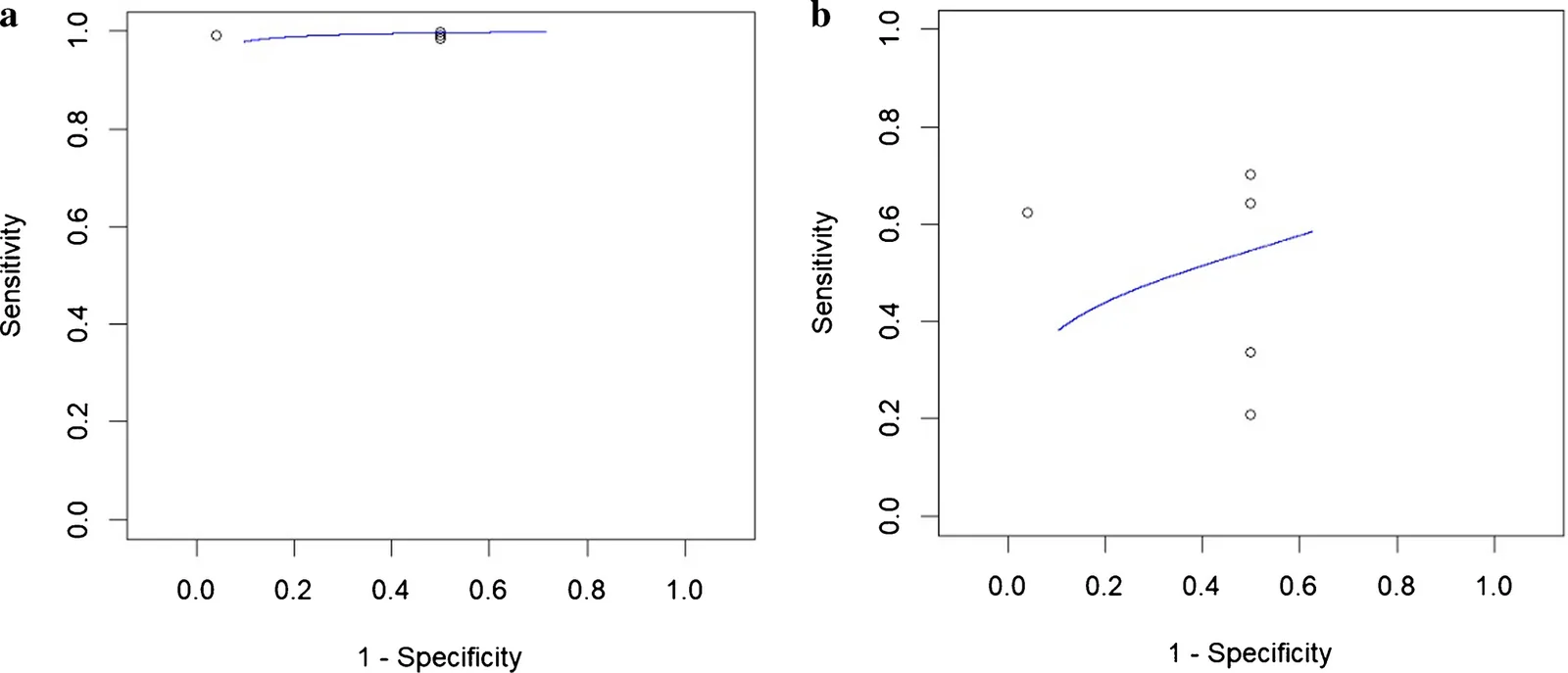
Summary receiver operating characteristics curve on the performance of [^18^F]-mFBG PET/CT (**a**) and [^123^I]-MIBG SPECT/CT (**b**) in neuroblastoma on lesion based analysis

SROC curves showed an optimal diagnostic performance of [^18^F]-mFBG PET/CT and a moderate diagnostic performance of [^123^I]-MIBG SPECT/CT (Fig. 8).

In three studies [16–18], a direct comparison using standardized scoring systems like the Curie score was executed and [^18^F]-mFBG PET/CT demonstrated higher scores.

## Discussion

In our systematic review and meta-analysis, we aimed to investigate the diagnostic performance of [^18^F]-mFBG PET/CT in NB patients compared to [^123^I]-MIBG SPECT/CT. [^18^F]-mFBG PET/CT is an emerging tool that has gained significant attraction as a non-invasive method to detect disease localizations in NB. However, its potential in the mere diagnostic evaluation is only preliminarily analyzed [19] and more solid data are shareable. In this article, we concentrated all the available evidence about sensitivity and specificity of [^18^F]-mFBG PET/CT compared with the more commonly and universally considered gold standard [^123^I]-MIBG scintigraphy.

[^123^I]-MIBG SPECT/CT is routinely used in the initial evaluation and follow-up of patients with NB but [^123^I]-MIBG SPECT/CT imaging procedure is relatively complex. It requires a 2-day schedule, with the radiopharmaceutical injected on the first day and imaging performed approximately 24 h later and sometimes also after 48 h [20]. The incorporation of the [^123^I] isotope makes thyroid prophylaxis with supersaturated potassium iodide recommended before the radiotracer injection. [^123^I]-MIBG planar and SPECT/CT scans are usually quite long and time-consuming, lasting 1–2 h, and for these reasons may necessitate sedation in pediatric patients. An additional disadvantage is the lower resolution power and less well-validated uptake semiquantitative parameters compared with PET/CT.

Previous in-human experiences showed that [^18^F]-mFBG is safe, presents favorable pharmacokinetics (faster and greater contrast of lesion detection) and has a comparable distribution to [^123^I]-MIBG [21, 22]. Moreover, it may avoid some of the limitations of [^123^I]-MIBG (such as the need for 2-day imaging and administration of iodine for thyroid blockade). Finally, the growing availability of new-generation PET scanners with a long axial field of view enables scanning these patients with minimal radiopharmaceutical activity and thus with a sharply reduced dose.

Our qualitative and quantitative research showed that there are no significant differences in terms of specificity among [^18^F]-mFBG PET/CT and [^123^I]-MIBG SPECT/CT in NB patients, while the difference in terms of sensitivity was significant, both at PBA and LBA. Particularly, the number of FN reports with [^123^I]-MIBG SPECT/CT is significantly higher than that with [^18^F]-mFBG PET/CT. In most cases additional sites recognized by PET/CT were bones followed by soft-tissue lesions [14–18]. Concerning bones, the increased site detection with [^18^F]-mFBG PET/CT was noted across multiple osseous segments, including both the axial and appendicular skeleton [17]. This difference may be related to the different resolution powers of PET/CT tomograph than SPECT/CT (especially if digital PET scanners are used) and the better target to background ratio with [^18^F]-mFBG.

Consequently, visual scores like Curie score criteria may be affected by the tracer. For example, Pandit-Taskar et al. [18] derived a higher Curie score in 72.5% of cases. Three [16–18] of the included studies performed a direct comparison using standardized scoring systems like the Curie score. However, it should be noted that the whole-body nature of PET/CT naturally overcomes the field-of-view limitations of regional SPECT, potentially leading to more accurate risk stratification.

The subsequent step should be to clarify the real impact of [^18^F]-mFBG PET/CT in the disease management of NB patients. Until now, it seems difficult to answer this question because the data available are not clear and complete, but we can hypothesize that the superiority of [^18^F]-mFBG PET/CT compared to [^123^I]-MIBG SPECT/CT in detecting NB lesions could help to stage more precisely the NB patients guiding the subsequent treatment.

Although the *I*^2^ value in our analysis suggested low statistical heterogeneity, the small number of studies included reduced the statistical power of this parameter. We recognize significant clinical heterogeneity regarding patient age (ranging from infants to adults) and technical protocols, which may affect the generalizability of our findings.

Beyond superior sensitivity, [^18^F]-mFBG PET/CT offers significant practical advantages. The entire procedure can be completed within 2–3 h, eliminating the need for a second-day scan and potentially reducing or avoiding sedation in children. The absence of free radioactive iodine removes the requirement for thyroid blockade. When combined with modern digital PET/CT or long-axial FOV systems, these factors contribute to a faster, more patient-friendly, and potentially lower-radiation-dose protocol, which is a critical consideration in pediatric oncology.

Furthermore, we would like to highlight that head-to-head comparisons of molecular imaging techniques employing different tracers, when based solely on diagnostic performance, represent only the starting point of a broader and more comprehensive evaluation. It is essential to recognize that diagnostic performance does not equate to clinical effectiveness: in other words, higher accuracy does not automatically translate into improved patient outcomes. The impact of a diagnostic test should be assessed across multiple domains, including technical characteristics, availability, diagnostic performance, influence on clinical decision-making, effects on patient outcomes, and cost-effectiveness. All these dimensions should be taken into account when comparing methods using different radiotracers in NB [11].

Beyond diagnostic performance, radiation dosimetry is a crucial factor in pediatric oncology. [^123^I]-MIBG SPECT/CT and [^18^F]-mFBG PET/CT fall into different dose classes due to their physical properties and administration protocols. For a standard administration, the effective dose for [^123^I]-MIBG is approximately 0.013 mSv/MBq, resulting in an average effective dose of roughly 3–5 mSv for a 5-year-old child (excluding the CT component). In contrast, [^18^F]-mFBG has shown a more favorable dosimetric profile, with an effective dose of approximately 0.024 mSv/MBq. However, due to the higher sensitivity of PET scanners, lower activities can be administered (e.g., 2–4 MBq/kg for [^18^F]-mFBG vs 11 MBq/kg for [^123^I]-MIBG), leading to a reduction in the total effective dose to approximately 1.5–2.5 mSv. Furthermore, the absence of thyroid-blocking agents—required for [^123^I]-MIBG to prevent free iodine uptake but unnecessary for the stable C-F bond in [^18^F]-mFBG—represents a clinical advantage and reduces the risk of unnecessary radiation to the thyroid gland.

While our meta-analysis highlights the superior diagnostic accuracy of [^18^F]-mFBG, its clinical implementation faces significant logistical hurdles. Currently, the worldwide distribution of gamma-cameras remains significantly higher and more uniform than that of PET/CT tomographs, particularly in developing countries or non-academic centers. Moreover, [^123^I]-MIBG is a commercially available tracer with a well-established supply chain, whereas [^18^F]-mFBG is still largely produced in centers with on-site cyclotrons or specialized radiopharmacies. This discrepancy suggests that while [^18^F]-mFBG may become the preferred method in tertiary referral centers, [^123^I]-MIBG SPECT/CT will likely remain the backbone of neuroblastoma staging in many regions for the foreseeable future due to the existing infrastructure and lower costs associated with SPECT technology.

In this regard, an advantage of [^123^I]-MIBG SPECT/CT compared to [^18^F]-mFBG PET/CT remains its wider availability, conversely, a higher diagnostic performance of [^18^F]-mFBG PET/CT compared to [^123^I]-MIBG SPECT/CT has been demonstrated by our meta-analysis. Technical aspects seem also in favour of [^18^F]-mFBG PET/CT. Future studies should assess differences among these imaging methods in terms of change of management, effects on patient outcomes and cost-effectiveness.

A critical aspect regarding the clinical adoption of [^18^F]-mFBG is its role in selecting patients for [^131^I]-MIBG therapy. Currently, [^123^I]-MIBG remains the standard “theranostic” companion to predict uptake for therapy and help in the selection of patients before radionuclide therapy. While [^18^F]-mFBG shows higher sensitivity, lesions detected only on PET but not on [^123^I]-MIBG might lack sufficient expression of the norepinephrine transporter to ensure therapeutic benefit from [^131^I]-MIBG. Therefore, while [^18^F]-mFBG is superior for staging and response assessment, a confirmatory [^123^I]-MIBG scan may still be required before starting radiometabolic therapy, at least until further data on the predictive value of PET-derived parameters for [^131^I] treatment are available.

The main limitations of our systematic review and meta-analysis include the low number of included studies (*n*=5) and the subsequent small sample analyzed, also due to the rarity of disease considered and the novelty of the radiopharmaceutical [^18^F]-mFBG, and the heterogeneity of patients, disease characteristics, and treatment. Other limitations were the high rate of positive PET/CT compared to negative ones and the lack of histopathological confirmation of all lesions analyzed, which was not feasible for all sites. Usually other imaging examinations and clinical follow-up was taken as reference for the final diagnosis. Furthermore, the studied populations had a high disease prevalence, resulting in a very low number of true negative (TN) cases (*n*=11). This small TN pool makes the pooled specificity estimate (82%) unstable, as evidenced by the wide confidence intervals (53–95%). The specificity results should therefore be interpreted with caution. The lesion-based analysis may be subject to clustering effects, as multiple lesions within the same patient are not independent. However, lesion base analysis provides a more global view of the tracer’s detection capability across different anatomical sites.

Besides, our study is limited by the inherent incorporation bias in the included literature, where the index test ([^123^I]-MIBG) often contributes to the reference standard definition. This is a common challenge in pediatric nuclear medicine trials where histopathological confirmation of all lesions is not possible.

Future studies should include more patients in remission or with low disease burden to better define the specificity of both tracers.

Another limitation is the current investigational status of [^18^F]-mFBG. While its commercial availability in North America and parts of Europe is currently limited, the consistent superiority in sensitivity demonstrated across international cohorts suggests that [^18^F]-mFBG has the potential to redefine the gold standard. The transition from single-photon tracers to PET-based catecholaminergic imaging follows a broader trend in nuclear medicine, where superior resolution and patient convenience eventually drive infrastructure changes and commercial distribution.

On the other hand, one strength of the studies available in the literature is the prospective design of the research. Also another strength of the meta-analysis is the strict selection of articles (only those performing head-to-head analysis).

## Conclusions

With limitations of the heterogeneity of the studies included, this systematic review and meta-analysis demonstrated that [^18^F]-mFBG PET/CT shows a significant superiority in sensitivity over [^123^I]-MIBG SPECT/CT for studying NB both at PBA and LBA, while maintaining comparable specificity. [^18^F]-mFBG PET/CT could be considered in the future as a potential replacement of [^123^I]-MIBG SPECT/CT as the imaging gold standard due to its superior diagnostic performance and logistical advantages (faster and comfortable protocol, no thyroid blockade, no need of sedation, higher resolution power). However, its routine clinical implementation will depend on larger prospective trials, cost-effectiveness analyses, and wider tracer availability.

## Supplementary Information

Below is the link to the electronic supplementary material.

ESM 1 Supplementary Material 1 (DOCX 206 KB)

## Data Availability

No datasets were generated or analysed during the current study.
